# Circumventing thermodynamics to synthesize highly metastable perovskites: nano eggshells of SnHfO_3_†

**DOI:** 10.1039/d2na00603k

**Published:** 2022-11-08

**Authors:** Eric A. Gabilondo, Ryan J. Newell, Jessica Chestnut, James Weng, Jacob L. Jones, Paul A. Maggard

**Affiliations:** Department of Chemistry, North Carolina State University Raleigh NC 27695 USA paul_maggard@ncsu.edu; Department of Materials Science and Engineering, North Carolina State University Raleigh NC 27695 USA; X-Ray Sciences Division, Argonne National Laboratory Lemont IL 60439 USA

## Abstract

Sn(ii)-based perovskite oxides, being the subject of longstanding theoretical interest for the past two decades, have been synthesized for the first time in the form of nano eggshell particle morphologies. All past reported synthetic attempts have been unsuccessful owing to their metastable nature, *i.e.*, by their thermodynamic instability towards decomposition to their constituent oxides. A new approach was discovered that finally provides an effective solution to surmounting this intractable synthetic barrier and which can be the key to unlocking the door to many other predicted metastable oxides. A low-melting KSn_2_Cl_5_ salt was utilized to achieve a soft topotactic exchange of Sn(ii) cations into a Ba-containing perovskite, *i.e.*, BaHfO_3_ with particle sizes of ∼350 nm, at a low reaction temperature of 200 °C. The resulting particles exhibit nanoshell-over-nanoshell morphologies, *i.e.*, with SnHfO_3_ forming as ∼20 nm thick shells over the surfaces of the BaHfO_3_ eggshell particles. Formation of the metastable SnHfO_3_ is found to be thermodynamically driven by the co-production of the highly stable BaCl_2_ and KCl side products. Despite this, total energy calculations show that Sn(ii) distorts from the A-site asymmetrically and randomly and the interdiffusion has a negligible impact on the energy of the system (*i.e.*, layered *vs.* solid solution). Additionally, nano eggshell particle morphologies of BaHfO_3_ were found to yield highly pure SnHfO_3_ for the first time, thus circumventing the intrinsic ion-diffusion limits occurring at this low reaction temperature. In summary, these results demonstrate that the metastability of many theoretically predicted Sn(ii)-perovskites can be overcome by leveraging the high cohesive energies of the reactants, the exothermic formation of a stable salt side product, and a shortened diffusion pathway for the Sn(ii) cations.

## Abstract 2

Through a multifaceted synthetic technique and leveraging surface diffusion, a new Sn(ii)-perovskite, SnHfO_3_, has been synthesized as nano eggshells for the first time.

## Graphical abstract

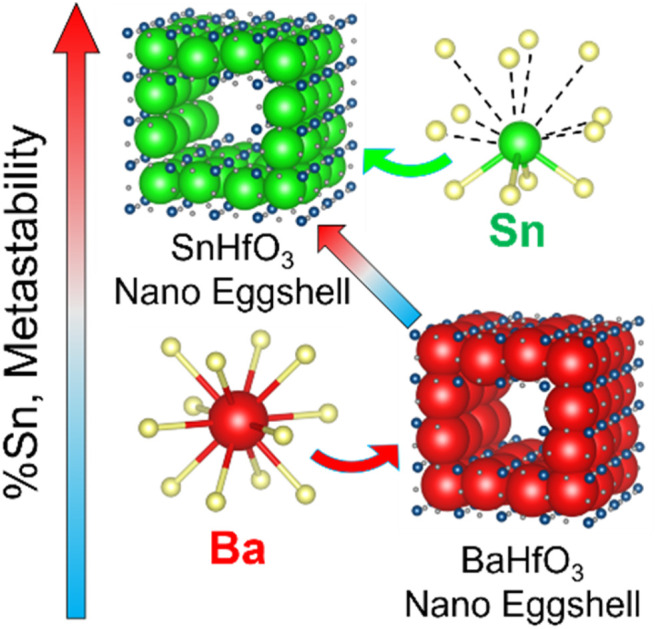

## Introduction

Metal oxides are widely regarded for the tuneability of their physical properties that scale with the complexity of their compositions and structures. Among complex metal oxides, metastable compounds have garnered keen recent interest for their potential high technological impact, such as for ferroelectrics, ultrahard materials, and in semiconducting photocatalysts. Metastable materials constitute an elusive and dynamic frontier due to the synthetic challenges of kinetic stabilization, which is usually absent in conventional solid-state synthetic approaches at high temperatures.^1–3^ Low-temperature ‘Chimie Douce’ techniques have been effectively employed in the crystallization of some thermodynamically unstable oxides.^4^ Most recently, topotactic ion-exchange reactions, such as mediated by a low-melting salt flux, have emerged as a potent tool in the synthesis of metastable oxides with close-packed structures.^5–7^

Synthetic challenges to attain metastable solids have been exemplified by the pursuit of Sn(ii)-based perovskite oxides over the past two decades, as motivated by their predicted properties as Pb(ii)-free piezoelectrics or as semiconducting photocatalysts. For example, Sn(ii)-based perovskites (*e.g.*, Sn(Zr_1−*x*_Ti_*x*_)O_3_) have been predicted to exhibit greater electric polarization as compared to Pb(ii)-perovskites (*e.g.*, Pb(Zr_1−*x*_Ti_*x*_)O_3_; PZT) in addition to their reduced toxicity.^8–12^ Despite their promising potential, synthetic pathways to these thermodynamically unstable materials remain unsuccessful. As a conventional reagent, binary SnO rapidly oxidizes or disproportionates at temperatures as low as ∼250 °C.^13^ Furthermore, Sn(ii)-oxides are generally susceptible to thermal decomposition at typical reaction temperatures as a result of their thermodynamic instability to yield the simpler oxides. For example, a recent report from the Maggard and Jones groups has shown that related metastable perovskites decompose beginning at only ∼350–400 °C into the simpler constituent oxides. Thus, a deeper understanding of the fundamental factors governing synthesizability of metastable Sn(ii)-oxides is of critical importance to synthesize Sn(ii)-containing perovskites. Some reports suggest that a synthesizable metastable phase of a given composition must occur within ∼100–200 meV atom^−1^ above the convex hull,^14–16^ but these synthetic limits remain relatively poorly explored.

Previous work from our research group has further investigated these assertions by demonstrating the synthesis of Sn(ii)-rich perovskites *via* low-temperature ion-exchange techniques. In the SnMO_3_ perovskite systems (M = Ti(iv), Zr(iv), Hf(iv), Sn(iv)), for example, it was hypothesized that their synthesizability could be significantly increased by (a) maximizing the lattice cohesive energy of the underlying MO_6_ substructure and (b) targeting composition spaces with few competing lower-energy polymorphs. Additionally, these reports highlighted the critical role of temperature in both the formation and decomposition of the metastable phase *via* ion-diffusion mechanisms. In all prior cases however, a pure Sn(ii)-perovskite remained unattainable with the maximum achievable cation-exchange limit straddling ∼60–70% Sn(ii)-substitution before onset of significant phase decomposition.

Thus, in an effort to further drive the limits of metastability in Sn(ii)-containing perovskites, we herein have employed a multi-faceted ‘Chimie Douce’ technique which leverages a low melting salt, the co-formation of stable side products, as well as nano eggshell morphologies. The BaHfO_3_ perovskite was chosen as the starting reactant for the synthesis of a model SnHfO_3_ perovskite owing to its high cohesive energy (as exemplified by the high melting point for BaHfO_3_ of ∼2620 °C) and the absence of competing lower-energy polymorphs such as the ilmenite or rutile structures.^17,18^ Previously reported synthetic methodologies to synthesize Sn(ii)-containing oxides have consistently involved the use of a relatively ‘hard’ peritectic SnCl_2_/SnF_2_ flux (m.p. ∼220 °C), *e.g.* SnClF. As the fluoride anion can also function as a mineralizer,^19^ and irreversibly dissolving the product at high loadings,^48^ a new low-melting salt was identified as a flux reagent. The relatively ‘softer’ and lower-melting chloride-based flux, a KCl/SnCl_2_ eutectic, (36.5/63.5 mol%, m.p. ∼180 °C) or KSn_2_Cl_5_, was thus investigated at a remarkably lower reaction temperature of ∼200 °C for driving the formation of the resulting metastable SnHfO_3_ perovskite. This has resulted in its first reported successful synthesis with 100% A-site Sn cations, achieving its maximum metastability, and thus paving the way to the synthesis of many theoretically predicted Sn(ii) perovskites.

## Experimental methods

### A. Synthesis of BaHfO_3_ and Sn(ii)-exchange

Micron-scale BaHfO_3_ particles were synthesized by the ceramic method as described in previous work.^5^ BaHfO_3_ hollow nanoparticles were synthesized *via* hydrothermal route similar to prior reports.^20,21^ Ba(NO_3_)_2_ (Baker, 99.9%) and HfCl_4_ (Acros Organics, 99%) in a 1.1 : 1 ratio were suspended in ∼5 mL of ethanol. For every 1 g of reagents loaded, 20 mL of concentrated 12 M or 16 M KOH solution was then added dropwise to the mixture and allowed to stir for 1 h. The slurry was transferred to a Teflon-lined stainless-steel autoclave and reacted at 200 °C for 24 h. The powder product was then washed with 150 mL of water, followed by 100 mL of dilute acetic acid, then another 150 mL of water before drying at 80 °C. The 12 M KOH product was then ground and annealed in air at 1000 °C for 2 h yielding ∼350 nm hollow particles, whilst the 16 M KOH product yielded ≤ 150 nm nano eggshells. Yield was usually ∼90 wt% regardless of scale.

Nanoshells of the Sn(ii)-hafnate perovskites were synthesized by reacting the BaHfO_3_ (*i.e.*, the micron sized, or ∼350 nm hollow particles or <150 nm nano eggshell morphologies) with a KSn_2_Cl_5_ salt melt. This KSn_2_Cl_5_ flux (m.p. ∼180 °C) was first made by grinding 0.365 mol of KCl (Fischer, >99.5%) with 0.635 mol SnCl_2_ (Alfa Aesar, 99.5%) under Ar until homogeneous. A 10-fold molar excess of the mixture was added to the three different BaHfO_3_ particles and was ground intimately with a mortar and pestle under Ar, typically not exceeding ∼0.75 g total mass. The homogenized powders were loaded into an evacuated fused-silica ampoule and reacted in a muffle furnace at 200 °C for 36 h and allowed to radiatively cool. The product was washed in 200 mL of water followed by 100 mL of ethanol then dried at 80 °C overnight yielding a faint-yellow powder. Yield was usually ∼90 wt%. High-purity SnHfO_3_ was prepared by using this method, as judged by powder XRD and EDS data, on the as-prepared ≤ 150 nm nanoparticles.

### B. Bulk characterization

Experimental powder X-ray diffraction (XRD) was measured on a Rigaku R-Axis Spider using a sealed X-ray CuKα (*λ* = 1.54056 Å, 40 kV, 36 mA) source in the Debye–Scherrer geometry with a curved image-plate detector. UV-Vis diffuse reflectance spectra (DRS) of (Ba_1−*x*_Sn_*x*_)HfO_3_ were collected on a Shimadzu UV-Vis-NIR spectrophotometer (UV-3600) equipped with an integrating sphere detector with 200–1500 nm range and flat BaSO_4_ (Alfa Aesar, 99%) surface served as the background reference. A commercially available SnO_2_ (Alfa Aesar, 99.9%) was used as a standard. The analyte was evenly spread and pressed onto the background reference and reflectance of the analyte was recorded and transformed using the Kubelka–Munk remission function and plotted as a Tauc plot *versus hν*.^22^ Linear interpolation of the transformed absorption band edge resulted in the approximate direct and indirect bandgaps.^23^ Raman spectroscopy was performed on a Horiba XploRA PLUS equipped with a Horiba Scientific CCD detector and a 532 nm excitation laser. The hole size, slit size, and grating were 500 μm, 200 μm, and 400 nm, respectively, and a 1 percent filter was applied to avoid oxidation during the measurements. The signal was acquired over 10 accumulations of 10 seconds.

High energy X-ray measurements were taken at 105.7 keV at beamline 11-ID-C at the Advanced Photon source. A Pilatus 2M CdTe detector was used with detector threshold parameters set at 105.7/50 keV. A gain map was collected for the detector at 105.7 keV immediately before the measurement. Sample to detector distance was 300 mm and a series of 1 second exposures were taken for a period of 60 seconds.

### C. Electron microscopy and energy dispersive spectroscopy

High resolution images and elemental analyses of (Ba_1−*x*_Sn_*x*_)HfO_3_ were performed on a JEOL 6010LA scanning electron microscope (SEM) with an accelerating voltage of 20 kV. A JEOL EDXS silicon drift detector was used to determine elemental composition. Nanoscale resolution images were acquired using a ThermoFisher Talos F200X with 200 kV accelerating voltage. Energy dispersive spectroscopy (EDS) spectra were collected using a 200 pA beam with a Super-X EDS detector and reported as atomic percentages.

### D. Total energy calculations

Total internal energy calculations were used to estimate the stability of SnHfO_3_*versus* the decomposition to binary oxides SnO and HfO_2_ using previous methods,^5^ which were benchmarked to formation energies at 0 K in the Open Quantum Materials Database (OQMD) using VASP.^24–28^ To probe the nanoshell arrangement of SnHfO_3_ interfaced to BaHfO_3_, as found experimentally (described below), total internal energies were calculated using density functional theory in the Vienna *Ab Initio* Simulation Package (VASP; ver. 4.6) for a layered configuration and for models that simulate the increasing interdiffusion of Sn/Ba cations across a SnHfO_3_–BaHfO_3_ interface. First, a 2 × 2 × 8 super structure of the cubic perovskite structure was created, with the first four perovskite layers having the SnHfO_3_ composition and the next four perovskite layers having the BaHfO_3_ composition. Sequential models were then also created that shifted two of the Ba/Sn cations at a time, representing intermediate atomic configurations between the fully segregated SnHfO_3_–BaHfO_3_ and fully mixed (Ba_1−*x*_Sn_*x*_)HfO_3_ structures, resulting in a total of 2, 4, 6, and 8 (fully mixed) cation displacements. For each of these 5 superstructure models, Perdew–Burke–Ernzerhof functionals were used within the generalized gradients approximation. The structures were first geometry relaxed, with 10^−5^ and 10^−2^ as the convergence criteria for the total energy and ionic steps, respectively. The Brillouin-zones for each were automatically sampled using a 4 × 4 × 1 *Γ*-centered *k*-point grid for the geometry relaxation and dispersion force corrections were applied within the DFT-D3(BJ) scheme and Becke–Johnson damping.

## Results and discussion

### A. Synthesis methodology and principles of cation exchange

Cation exchange reactions performed at low temperatures, without dissolving the underlying substructure, are typically limited by the slow ion-diffusion through their crystalline structures. The Sn(ii)-exchange appears to be severely ion-diffusion limited at a given temperature when a soft flux is used, *i.e.*, the perovskite substructure is not dissolved. Regardless of the particle size, the thermodynamics of the reaction would be expected to remain constant. Thus, two possible routes to achieve a fully exchanged Sn(ii)-perovskite, *i.e.*, SnMO_3_, would be to, (1) increase the reaction temperature for greater ion-diffusion, or (2) decrease the required diffusion lengths of the Sn(ii) cations within the particles. For highly metastable oxides, the former approach typically results in decomposition, especially as Sn(ii)-oxides decompose as low as only ∼350 °C. Therefore, modification of the particle sizes and morphologies was investigated to probe the feasibility of the latter approach, while also maintaining crystallinity.

The BaHfO_3_ precursor was prepared as both micron-sized particles using high-temperature methods, and in two different nano eggshell morphologies with varying shell thicknesses and particle sizes *via* a low-temperature hydrothermal approach. This hydrothermal synthetic technique has been demonstrated by rigorous TEM to form crystalline BaHfO_3_ as highly uniform nano eggshell morphologies with particle thickness and diameters based on the basicity of the aqueous media. The particles obtained by this synthetic route were determined to be ∼350 nm in diameter with ∼60–70 nm thick shells when prepared with 12 M KOH, and as ≤150 nm in diameter with ∼20 nm thick shells when prepared using 16 M KOH. By XRD, all BaHfO_3_ sizes fit well with the known *Pm*3̄*m* perovskite structure (Fig. S1A, C and E†). The ∼150 nm crystallites have significant shifting to lower 2*θ* from a doubling of the unit cell (∼8.38 Å), which is ascribed to numerous defects common to nanoparticle crystallinity. Thus, these three different particle morphologies were used to interrogate the relationships between particle sizes and Sn(ii) cation diffusion limits by synthetically targeting the predicted, metastable SnHfO_3_ perovskite, and leading to the discovery of a new route to prepare many other predicted Sn(ii)-based perovskites.

**Fig. 1:**
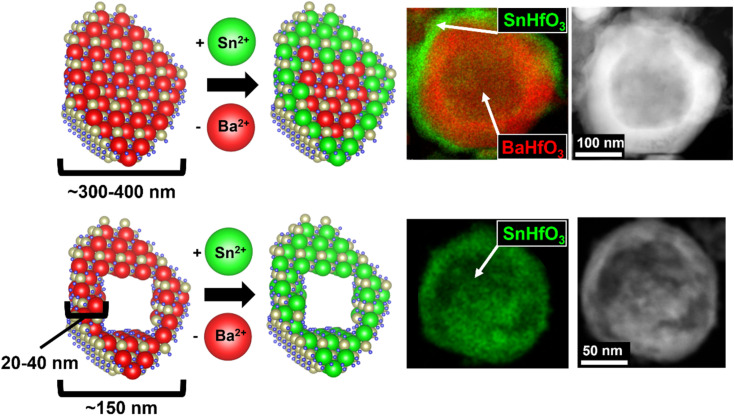
Schematic representation of soft flux-based Sn(ii)-exchange (left) with example TEM/EDS images (right). The hollow nano eggshell BaHfO_3_ are prepared in two different size regimes then reacted with the KSn_2_Cl_5_ flux at 200 °C. Thick-shelled, larger particles (top) react at the surface and are diffusion limited, resulting in a Sn-rich perovskite shell. Thin-shelled, smaller particles (bottom) react fully and Sn diffuses through the 20–40 nm shell, achieving full Sn(ii)-exchange.

The Sn(ii)-exchange reaction proceeded as follows, as also schematically described in Fig. 1 along with TEM/EDS snapshots that will be described in more detail further below. The various BaHfO_3_ products were reacted at a low reaction temperature of 200 °C within the KSn_2_Cl_5_ flux (m.p. ∼180 °C) and causing a Sn-for-Ba cation exchange to occur within the particles' surface regions. The overall reaction is exothermic and is thermodynamically driven by the exothermic formation of the BaCl_2_ and KCl salts from KSn_2_Cl_5_ (Δ*H*_f_ ≈ −527 kJ mol^−1^, −5.46 eV mol^−1^), *i.e.*, 2BaHfO_3_ + KSn_2_Cl_5_ → 2SnHfO_3_ + 2BaCl_2_ + KCl (Δ*H*_rxn_ ≈ −28 kJ mol^−1^, −0.29 eV mol^−1^), and thereby also yields the metastable SnHfO_3_. Both the large excess loading of KSn_2_Cl_5_ and the produced BaCl_2_ and KCl are highly soluble in water and are easily washed away, leaving the high purity perovskite product. Powder XRD data of these products and this reaction pathway are shown in Fig. S2.† Surface energies of the nano- and micron-scale particles were assumed to be negligible, as prior investigations have demonstrated that they are nearly equivalent to the bulk scale unless particle sizes are <∼2–5 nm, much smaller than the particles investigated herein.^29,30^ Further, the surface energy contribution is primarily critical in polymorphic phase transformations on the nanoscale when phase-transition barriers are small, yet negligible to the chemical transformations described above, *e.g.*, TiO_2_ rutile to brookite ∼0.71 kJ mol^−1^.^31,32^ Given the extremely small chemical diffusion coefficients of the Ba cation in the perovskite structure, of ∼10^−18^ to 10^−20^ cm^2^ s^−1^ at 1200 K, intrinsic cation diffusion would be expected to be severely restricted at this very low reaction temperature, forming a Sn(ii)-enriched hafnate perovskite at the surface. Thus, a comparison of the reactivity of micron-to nanometer-sized particles helps to assess the diffusional limitations. Increasing the required diffusion length to the micron-scale would be expected to minimize Sn(ii)-substitution (Fig. 1, top), while decreasing sufficiently small enough would potentially achieve a pure SnHfO_3_ perovskite (Fig. 1, bottom). While the smallest nanometer-sized BaHfO_3_ particles exhibits the shortest required diffusion lengths, these can also potentially enable a more facile decomposition.

### B. Bulk characterization

**Fig. 2:**
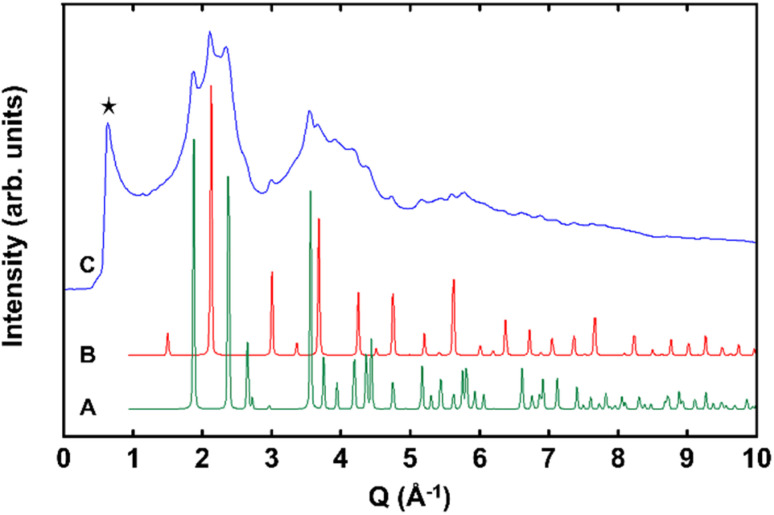
High-energy synchrotron XRD stack plot shows (A, green) simulated cassiterite SnO_2_*P4mmm*, (B, red) simulated SnHfO_3_ as a *Pm*3̄*m*, and (C, blue) the ∼150 nm SnHfO_3_ nano eggshell product. The low Q peak is denoted by a black star.

For micron-sized BaHfO_3_, the Sn(ii)-exchange reaction produced no discernible change in the powder X-ray diffraction (PXRD) data, or visually. The compound retained a cubic perovskite structure, consistent with previous reports on zirconate and titanate perovskites. Both nano eggshell products, however, turned a faint-yellow color after the Sn(ii)-exchange reaction. Further, after Sn(ii) exchange, the powder XRD of the ∼350 nm nanoshells showed no significant changes, while the ∼150 nm nano eggshell morphologies exhibited X-ray diffraction that grew largely diffuse in nature, albeit with retention of the primary perovskite reflections, *e.g.*, 110, 210, yet apparent (Fig. 1S†). To try and further resolve the perovskite structure, high-energy synchrotron XRD were collected on the ∼150 nm eggshell morphologies, shown in Fig. 2. The diffraction data are highly diffuse; however, the perovskite reflections as well as SnO_2_ are apparent. A thin surface layer of SnO_2_ is common in known Sn(ii)-oxides owing to the sensitivity of surface Sn(ii) cations to oxidation in air or water,^33,34^ which is likely exacerbated in the XRD in this case due to the large surface area of the nanoparticles. Additionally, a low *Q* peak is clearly visible at ∼0.67 Å^−1^. The low *Q* peak in conjunction with the diffuse scattering suggests the SnHfO_3_ nano eggshells exhibit some intermediate range ordering with long range structural disorder, similar to literature reports of glassy and amorphous solids such as zeolite-types and alkali silicates.^35–37^

**Fig. 3:**
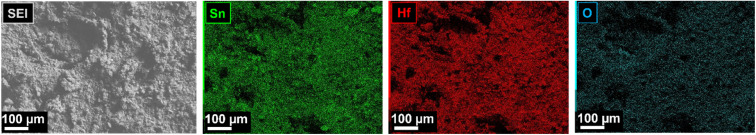
Representative SEM (left) and EDS mapping (right) of ∼150 nm SnHfO_3_ particles from bulk-scale. EDS maps show Sn in green, Hf in red, and O in blue. Ba was not detected (Fig. S9C†). Additional images and EDS spectra shown in ESI (S5–S8†).

Bulk-scale SEM/EDS were collected for each phase to determine the concentrations of Sn, given in Fig. S6–S9,† with a representative set of images shown in Fig. 3, demonstrating the bulk homogeneity of Sn and Hf in the ∼150 nm exchange product. The EDS spectra show that as the particle size decreases, the Sn : Ba ratio increases, and Ba was not detected in the ∼150 nm particles (Fig. S9†). Table 1 lists a summary of the quantitative EDS analysis results where the as-synthesized BaHfO_3_ precursors were used as an internal standard. These data suggest the soft Sn(ii)-exchange resulted in <10 mol% Sn-exchanged in the micron-sized particles. As the particles' sizes decreased to ∼350 nm and corresponding shell thickness to ∼60–70 nm, the concentration of Sn increased significantly up to ∼25 mol%. After further decreasing the particle size to ≤ 150 nm, only Sn, Hf, and O and a small amount of Cl were detected by EDS, signifying full exchange and the attainment of pure SnHfO_3_. It is estimated that the scale of the diffusion distance at 200 °C was on the order of tens of nanometers. These bulk results strongly support that the completeness of the Sn(ii)-exchange into the perovskite is governed by surface-terminated diffusional limitations.

**Table 1:** Tabulated results of EDS data for (Ba_1−*x*_Sn_*x*_)HfO_3_ as a function of the particle size following Sn(ii)-exchange with KSn_2_Cl_5_. Mol% of Ba and Sn were normalized to solid-state prepared BaHfO_3_ as an internal standard. EDS images and spectra are shown in Fig. S6–S9

Element	∼1 μm	∼350^a^ nm	∼150^b^ nm
Ba mol%	22.81	18.77	0.00
Sn mol%	2.19	6.23	25.00
*X*_exp_	0.09	0.25	1.00
Form. unit	Ba_0.91_Sn_0.09_HfO_3_	Ba_0.75_Sn_0.25_HfO_3_ (nanoshell-on-nanoshell)	SnHfO_3_ (nano eggshell)

a Shell thickness is ∼60–70 nm.

b Shell thickness is ∼20–30 nm.

The products were further characterized by Raman spectroscopy to probe for changes in the short-range order. The *Pm*3̄*m* BaHfO_3_ precursor has no first-order Raman active modes.^38^ The fully Sn(ii)-exchanged product however has been predicted in several studies of analogous perovskites to distort from the A-site due to its stereoactive lone-pair, generating new Raman-active vibrational modes.^39^ The Raman spectra of the BaHfO_3_ nanoshells (Fig. S3†) shows only peaks consistent with monoclinic HfO_2_, resulting from a small HfO_2_ impurity common from the hydrothermal synthesis method.^40,41^ The SnHfO_3_ nanoshells showed no significant Raman scattering. These data suggest a nondistorted SnHfO_3_ cubic perovskite structure, and the absence of a distortion arising from the Sn(ii) 5s2 lone pair.

The Sn(ii)-substitution is also expected to decrease the optical bandgap significantly, as has been predicted^42^ and shown experimentally,^43^ although a fully Sn(ii)-perovskite has yet to be measured. The Sn(ii)-containing hafnates were therefore analyzed by UV-Vis diffuse reflectance spectroscopy (DRS) to characterize the changes in their optical band gaps, plotted in Fig. S4† and listed in Table S1.† The nanoshell BaHfO_3_ optical absorption edge occurs at ∼5.6 eV, consistent with previous reports of the large bandgap semiconductor. The nanoshell-over-nanoshell SnHfO_3_-on-BaHfO_3_ particles showed two distinct absorption edges, one at ∼5.5 eV from the BaHfO_3_ core and the second at ∼3.1 eV from the SnHfO_3_ shell. The fully exchanged SnHfO_3_ product has only a single absorption edge at ∼3.4 eV, significantly lower by ∼2 eV than the BaHfO_3_ precursor and further demonstrative of full Sn(ii)-substitution. The optical absorptions of both Sn(ii)-exchanged hafnates were also compared to a SnO_2_ standard, since SnO_2_ was observed in the synchrotron XRD and is likely present in appreciable amounts on the particle surfaces. The standard had a sharp optical absorption of ∼3.7 eV, consistent with reports of ≤10 nm particles of SnO_2_,^44^ and significantly larger than the measured SnHfO_3_ phases. Further, optical bandgaps in the observed ∼3.1–3.4 eV regime are typically found *via* epitaxial growth-induced lattice strain^45^ or complete removal of Sn(ii)-oxidation states through high-temperature annealing.^46^

### C. Scanning transmission electron microscopy

**Fig. 4:**
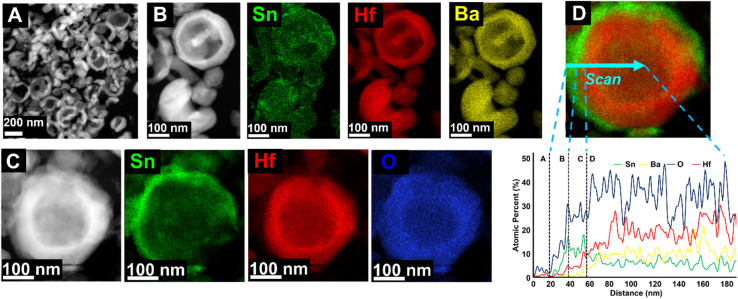
STEM images of ∼350 nm Sn(ii)-exchanged nanoparticles shown as HAADF micrographs (A–C) and EDS elemental mapping of accompanied by an EDS line scan of a representative particle (D). The EDS color maps are provided as Sn in green, Hf in red, Ba in yellow, and O in blue. The EDS line scan is given as atomic percentages measured across the blue arrow on the STEM micrograph at right (D).

Scanning transmission electron microscopy (STEM) accompanied by EDS was used to further investigate the individual nanoparticles' surfaces and compositions. Simple image processing software^47^ was used to estimate shell thicknesses based on the high-resolution images. Images and elemental mapping obtained for the ∼350 nm Sn(ii)-exchange products are shown in Fig. 4 along with an EDS line scan of a single representative particle. From the broader-scale images in Fig. 4(A and B), It can be seen the nanoparticles have hollow, cracked, and spherical morphologies. The cracking is likely an artifact of annealing the BaHfO_3_ precursor at 1000 °C, similar to prior studies on the high temperature annealing of hydrothermally-prepared metal oxides. The EDS mapping of a grouping of particles, Fig. 4B, as well as a single, whole, particle, Fig. 4C, showed that Sn is largely concentrated on the surfaces of the spheres producing a nanoshell-over-nanoshell SnHfO_3_-over-BaHfO_3_ morphology. An EDS line-scan, plotted in Fig. 4D, was performed across a representative particle to better resolve the compositional gradient. The first ∼20 nm of the scan is primarily the carbon support film; region A. Region B showed a Sn- and O-rich layer of ∼20 nm at the edge of the particle. This is likely because of the small amounts of surface oxidation (*i.e.*, SnO_2_) of the perovskite previously described. Region C shows a ∼20 nm layer consisting only of Sn, Hf, and O at a ratio of ∼2 : 1 : 5. This region overlaps with the Sn–O layer as well, likely giving a higher %Sn. The approximate composition in Region C is a mixture of SnHfO_3_ and SnO_2_, consistent with these observations. Region D is the remainder of the hollow particle of SnHfO_3_-over-BaHfO_3_ nanoshell-over-nanoshell which yields an average composition of ∼Ba_2/3_Sn_1/3_HfO_3_, or 1/3 SnHfO_3_ : 2/3 BaHfO_3_ and is consistent with bulk EDS. A small chloride incorporation of ≤5% was detected from the salt flux as a result of incomplete removal of the salt side products.

**Fig. 5:**
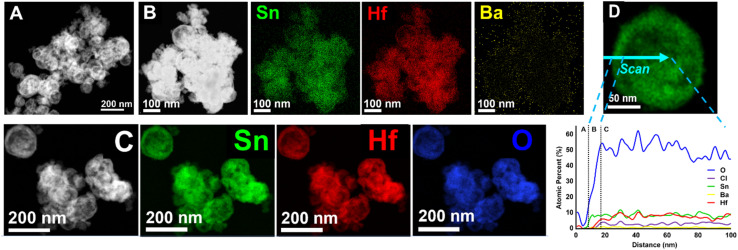
STEM images of ∼150 nm SnHfO_3_ nano eggshells shown as HAADF micrographs (A–C) and EDS elemental mapping of accompanied by an EDS line scan of a representative particle (D). The EDS color maps are provided as Sn in green, Hf in red, Ba in yellow, and O in blue. Ba was not detected by EDS. The EDS line scan is given as atomic percentages measured across the blue arrow on the STEM micrograph at right (D).

For comparison, Sn(ii)-exchange reaction of the BaHfO_3_ nano-eggshell morphologies produced 100% Sn(ii)-exchanged nano-eggshells, *i.e.*, SnHfO_3_ as shown similarly in Fig. 5. The particles are largely homogeneous and no larger than ∼150 nm. EDS data of a grouping of nanoparticles, Fig. 5(B and C) show only Sn, Hf, and O, and Cl, with no Ba detected. A similar EDS line-scan, Fig. 5D, through a representative particle shows the bulk particle (region C) contains Sn, Hf, and O at a ratio of ∼1 : 1 : 3 matching with a pure SnHfO_3_ composition. Region B shows a ∼20 nm Sn–O shell, similar to the surface oxidized layer of SnO_2_ as previously noted and observed in XRD. Region A is the carbon support film. A ≤5% Cl incorporation was also detected homogeneously throughout the particle. These results demonstrate clearly that surface cation diffusion limits have been circumvented in these soft Sn(ii)-exchange reactions using the nano-eggshell morphologies.

**Fig. 6:**
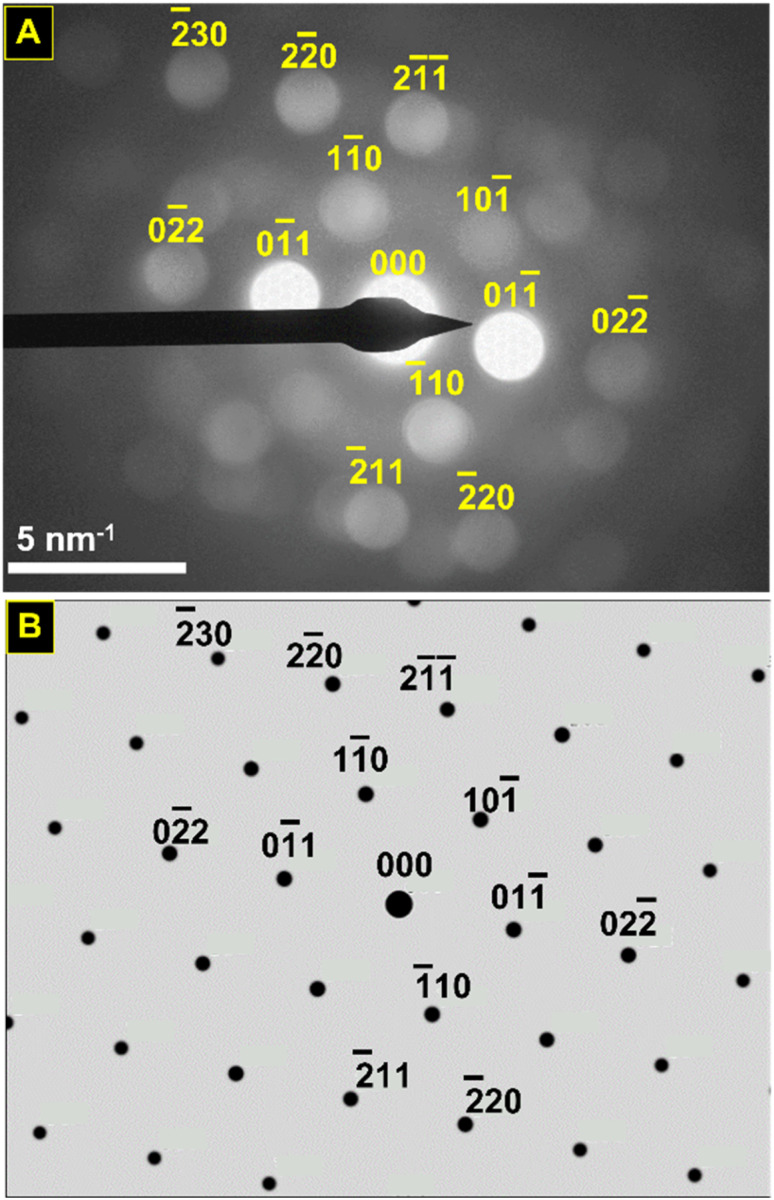
Convergent beam electron diffraction pattern of SnHfO_3_ nanoparticles (A) and simulated electron diffraction pattern for *Pm*3̄*m* SnHfO_3_ (*a* ≈ 4.18 Å) projected down the [111] axis (B). The ED beam is projected down the [111] axis, revealing diffuse diffraction in agreement with *Pm*3̄*m* symmetry, as evidenced by the {110} family (*d* ≈ 3 Å) and the {211} family, (*d* ≈ 1.75 Å).

Convergent beam electron diffraction (CBED) was performed on the nano eggshell morphologies SnHfO_3_ to further probe the crystalline nature, which showed very broad peaks generally consistent with perovskite structure. Owing to nanoparticle agglomeration, diffraction patterns obtained represented multiple orientations, shown in Fig. S5.†Fig. 6A shows a selected CBED pattern collected on a region of SnHfO_3_ along [111] zone axis with primary reflections consistent with a *Pm*3̄*m* perovskite structure with lattice constant ∼4.15–4.20 Å. The most intense {110} reflections correspond to *d*-spacing of ∼3.04 Å, and as compared to cubic BaHfO_3_ {110} *d*-spacing of ∼2.97 Å. Additionally the {211} reflections are apparent with *d*-spacings of ∼1.79 Å, in agreement with {211} *d*-spacings of ∼1.72 Å in BaHfO_3_. The CBED data along with the bulk- and nano-scale STEM demonstrate a retention of the cubic symmetry after full Sn(ii)-substitution and the synthesis of the first pure cubic Sn(ii)-perovskite in the form of stand-alone particles.

### D. Total energy calculations of Sn(ii)-diffusion

**Fig. 7:**
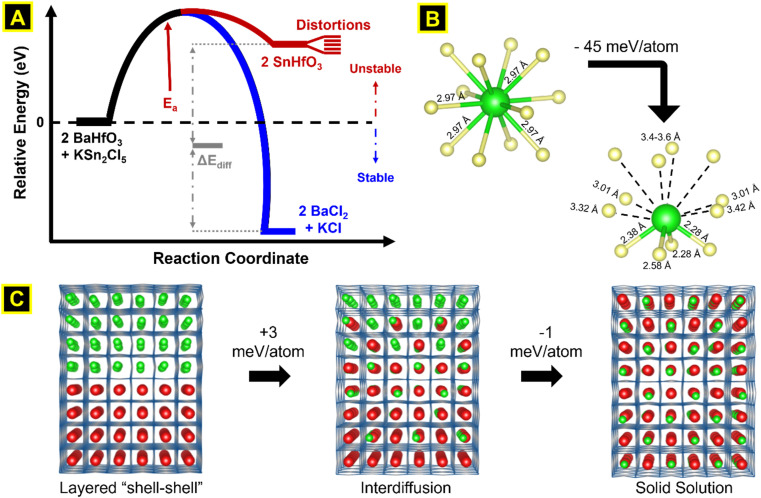
Schematic representation of reaction energetics (A), geometry relaxed Sn–O coordination (B), and supercells used to simulate interlayer Sn·Ba cation diffusion (C). The overall Sn(ii)-exchange reaction is exothermic (−0.29 eV mol^−1^), driven forward by the large heat of formation of BaCl_2_ and KCl *vs.* KSn_2_Cl_5_ (−5.46 eV mol^−1^). Upon relaxation, Sn undergoes an asymmetric distortion from the A-site with no preferential direction, lowering the internal energy (−45 meV per atom). Supercell relaxations of the SnHfO_3_–BaHfO_3_ layered interface and Sn(ii) diffusing stepwise across the layer shows the thermodynamic stabilizing effect of the shell–shell *versus* solid solution morphology is negligible (−2–3 meV per atom).

Electronic structure calculations were utilized to more deeply probe and understand the metastability of the SnHfO_3_ perovskite, both as stand-alone particles and as nanoshells over the BaHfO_3_ particles. Total energy calculations of the geometry-relaxed starting structures, starting from the idealized cubic *Pm*3̄*m* space group, were used to estimate the energetics of the Sn(ii) exchange reaction, as schematically represented in Fig. 7A. The SnHfO_3_ perovskite is metastable with respect to the binary oxides, *e.g.*, SnHfO_3_ → SnO + HfO_2_ (Δ*E*_rxn_ ≈ −646 meV per atom). The driving force of the reaction is thus the formation of the stable BaCl_2_ and KCl side products during the Sn(ii) exchange reaction, described above, which enables the net reaction to be overall exothermic by about −16 meV per atom. This however does not describe how its stability will be changed in the form of SnHfO_3_ nanoshells covering the BaHfO_3_ particles, and why nano eggshells might be more energetically favorable as compared to a fully mixed Sn/Ba solid solution model. Further, it is anticipated that the Sn(ii)-cation distortion, driven be lone-pair effects similar to Pb(ii)-perovskites, could potentially stabilize the SnHfO_3_ nanoshells.

To better understand the nanoshell SnHfO_3_ formation and diffusion limits, supercells of 8 × 2 × 2 dimensions of interfaced SnHfO_3_–BaHfO_3_ regions (green-red colors analogously in Fig. 7C) were constructed and geometry relaxed. For each model, the Sn(ii) cations were found to randomly distort with no preferential directionality and lowering the total energy by ∼44–46 meV per atom, as illustrated by the local coordination in Fig. 7B and the extended structure in 7C. The total energy of the nanoshell-over-nanoshell morphology was found to be −8.739 eV per atom. Two at a time, Sn and Ba atoms were randomly swapped then relaxed until these A-site cations and fully disordered, thereby simulating the progressive interdiffusion between the SnHfO_3_ layer and the BaHfO_3_ layer, Fig. 7C. After each step of Sn/Ba interdiffusion, the total energy increased, until a full interdiffusion of the Sn/Ba atoms had been reached in the supercell model. The resulting solid solution of fully mixed Sn/Ba sites was found to be ∼3.2 meV per atom higher in energy as compared to the nanoshell-over-nanoshell configuration. These energetic trends, though relatively small as compared to the impact of the distortion of Sn(ii) cations, are consistent with the formation of the nano eggshell morphologies of metastable SnHfO_3_. Therefore, the diffusional limits of the Sn(ii) cations were also found to stem from the absence of an energetic driving force for the full interdiffusion of the A-site cations, as well as the slow Ba/Sn diffusion rates at these low reaction temperatures. The SnHfO_3_ nanoshells were thus found to be somewhat stabilized as compared to A-site disordered configurations, yet kinetically stabilized against decomposition to the simpler oxides or alternatively polymorphs owing to the lack of sufficient thermal energy.

## Conclusion

In summary, a highly metastable Sn(ii) perovskite oxide, SnHfO_3_, has been synthesized for the first time in both a nanoshell-over-nanoshell and nano eggshell particle morphologies in high purity. The nanoshell morphologies effectively enable sufficient cation diffusion as well as kinetic stabilization against decomposition to simpler oxides. This was accomplished by using a soft ion-exchange technique, in which a low-melting point KSn_2_Cl_5_ flux was used to exchange Ba(ii) for Sn(ii) at BaHfO_3_ hollow nanoparticle surfaces, producing nano eggshell morphologies of SnHfO_3_ in high purity and yield. The structure was characterized by XRD, CBED, and spectroscopy and showed a retention of an overall, averaged, cubic perovskite structure. Geometry relaxation calculations indicate there is no energetically preferred Sn-displacement direction within the SnHfO_3_ nanoshells, as well as a negligibly small energetic preference for the formation of layered (*i.e.*, nanoshells) *versus* fully-disordered Sn/Ba regions of the particles. Thus, this work has demonstrated a new approach to circumvent the intrinsic barrier of ion-diffusion limits in low-temperature Sn(ii)-exchange reactions, and motivating further ongoing investigations into this material as well as other predicted Sn(ii)-based perovskite oxides.

## Author contributions

P. A. M. and J. L. J. supervised the project. E. A. G. designed and performed the synthesis experiments. R. J. N. performed STEM/EDS and CBED while E. A. G, and J. C. performed and analyzed the SEM/EDS. E. A. G. performed lab powder XRD structure analysis and J. W. performed high-energy synchrotron XRD. R. J. N collected Raman Spectroscopy and E. A. G. performed UV-Vis DRS measurements. P. A. M., E. A. G., J. L. J. and R. J. N. wrote the manuscript.

## Conflicts of interest

There are no conflicts to declare.

## Supplementary Material

NA-004-D2NA00603K-s001
