# Supplementary material for: Circumventing thermodynamics to synthesize highly metastable perovskites: nano eggshells of SnHfO3
Source: Nanoscale Adv. 2022 Nov 8;4(24):5320–9. doi: 10.1039/d2na00603k (PMC9724691; doi:10.1039/d2na00603k)
Supplement: NA-004-D2NA00603K-s001 [file NA-004-D2NA00603K-s001.pdf]

Electronic Supplementary Material (ESI) for Nanoscale Advances.
This journal is © The Royal Society of Chemistry 2022

# Supporting Information

Circumventing Thermodynamics to Synthesize Highly Metastable Perovskites: Nano Eggshells of $SnHfO_3$

Eric A. Gabilondo,[a] Ryan J. Newell,[b] Jessica Chestnut,[a] James Weng,[c] Jacob L. Jones[b] and Paul A. Maggard[a],*

[a] *Department of Chemistry, North Carolina State University, Raleigh, NC 27695-8204*
[b] *Department of Materials Science and Engineering, North Carolina State University, Raleigh, NC 27695-8204*
[c] *X-Ray Science Division, Argonne National Laboratory, Lemont, IL 60439*

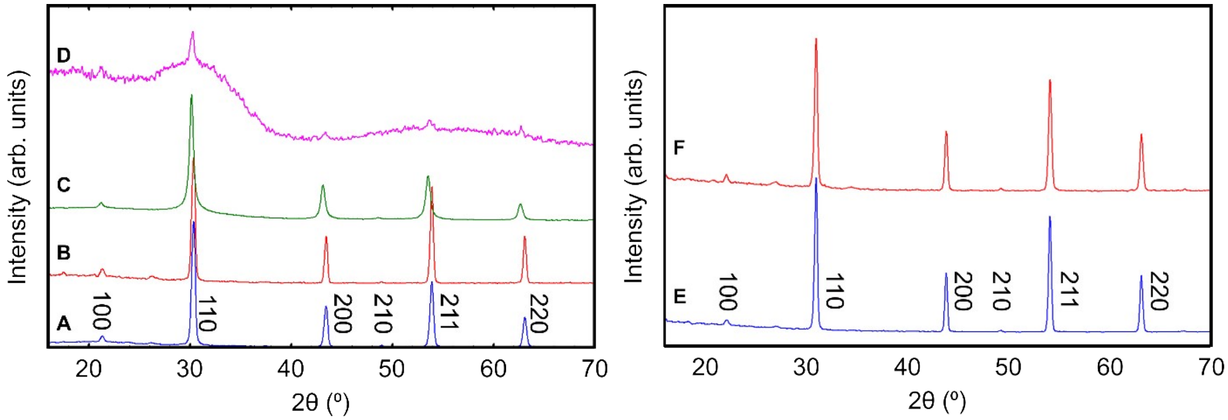


**Figure S1.** Powder XRD plots of (left) nano eggshell hafnates and (right) micron-sized hafnates before and after soft Sn(II)-exchange using the $KSn_2Cl_5$ molten flux. The left plot shows the nano eggshell ~350 nm $BaHfO_3$ (A, blue) before and (B, red) after Sn(II)-exchange and ~150 nm $BaHfO_3$ (C, green) before and (D, pink) after Sn(II)-exchange. The right plot shows the ~1 μm $BaHfO_3$ (E, blue) before and (F, red) after Sn(II)-exchange.

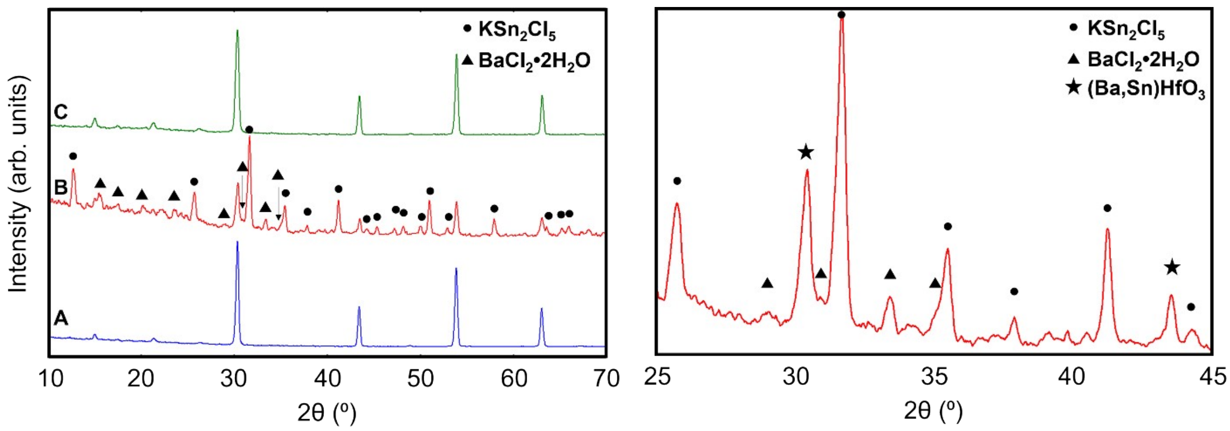


**Figure S2.** Powder XRD data for (A, blue) ~350 nm $BaHfO_3$ nano eggshells, (B, red) after reacting with a 10-molar excess of $KSn_2Cl_5$, and (C, green) after washing with water. The excess $KSn_2Cl_5$ and the $BaCl_2{\bullet}2H_2O$ exchange product are denoted with circles and triangles, respectively. The XRD data for the unwashed products (B) are shown focused on 25-45 $2\theta$ range to highlight the salt primary reflections.

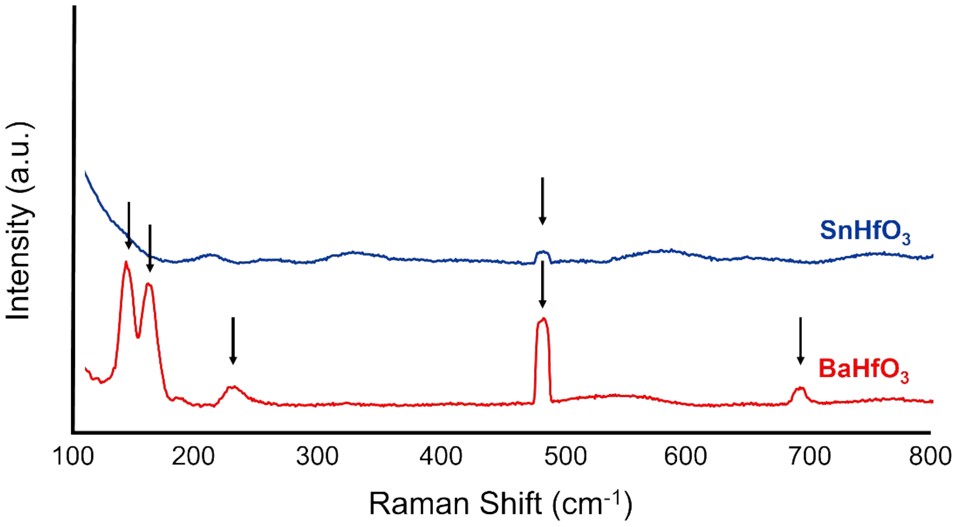


**Figure S3.** Normalized Raman spectra for $BaHfO_3$ precursor (bottom) and $SnHfO_3$ exchanged product (top) at 10% beam power. Peaks corresponding to $HfO_2$ impurity are labeled with arrows. The ~490 $cm^{-1}$ peak also has contribution from ambient lighting during measurement. No other Raman scattering is observed for either phase, consistent with the ideal cubic perovskite $Pm\bar{3}m$ symmetry.

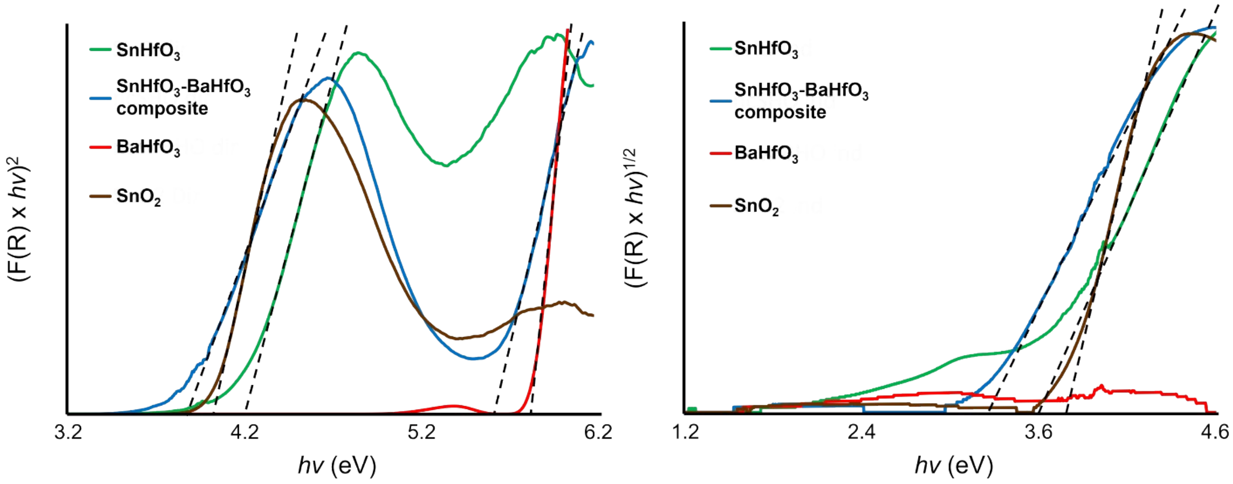


**Figure S4.** Representative UV-Vis diffuse reflectance data as a Tauc plot of (F(R) x *hv*)$^n$ with (left) n = 2 for direct transitions and n = ½ for indirect transitions. Curves are shown for $BaHfO_3$ precursor (red), the ~350 nm $SnHfO_3$-$BaHfO_3$ composite (blue), ~150 nm $SnHfO_3$ (green), and a commercial $SnO_2$ standard (brown). The $SnO_2$ standard confirms the change in optical absorption is primarily due to Sn(II)-substitution. Dashed lines trace where absorption edges were extrapolated to the baseline to estimate $E_g$.

**Table S1.** Tabulated direct and indirect optical bandgaps (Eg) obtained from Tauc plots as a function of Sn(II). A commercial $SnO_2$ standard is included.

| Mol Frac Sn(II) | Eg, direct (eV) | Eg, indirect (eV) |
|---|---|---|
| 0.00 | 5.7 | 5.6 |
| 0.25 | 3.8, 5.6 | 3.1, 5.5 |
| 1.00 | 4.2 | 3.4 |
| $SnO_2$ | 4.0 | 3.7 |

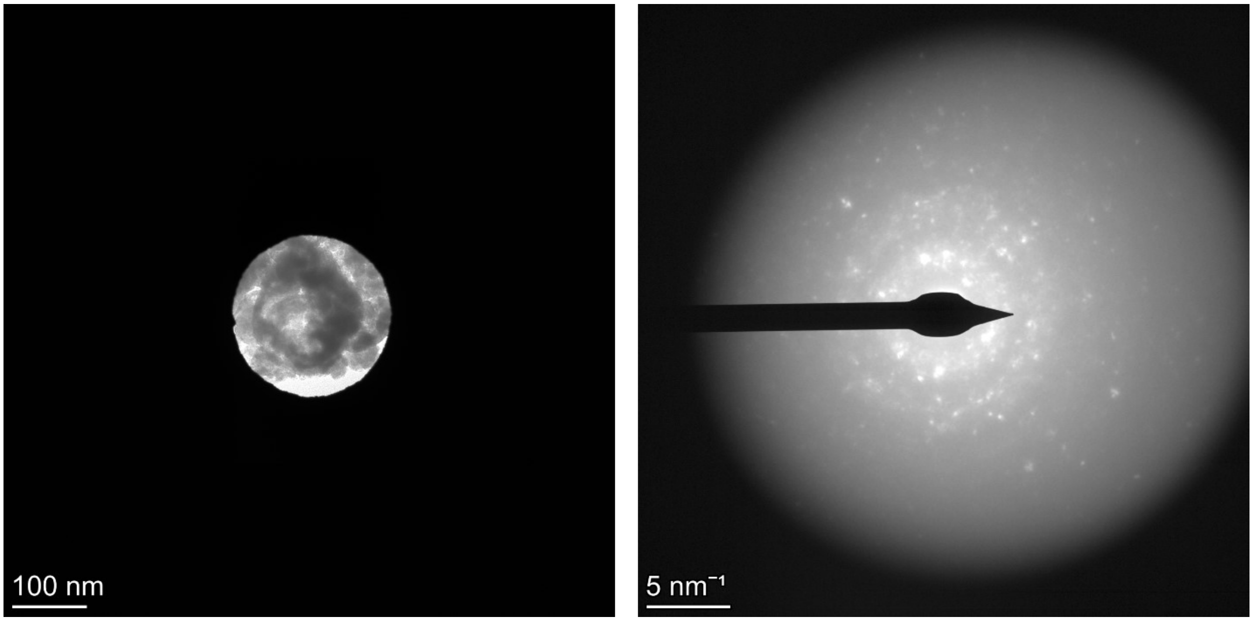


**Figure S5.** TEM micrograph with 10 nm SAED aperture and corresponding diffraction image, illustrating the polycrystalline nature in the agglomerated nanoparticles. As several orientations rendered SAED unproductive, CBED investigation was pursued to limit the interaction volume as much as possible, enabling structural determination.

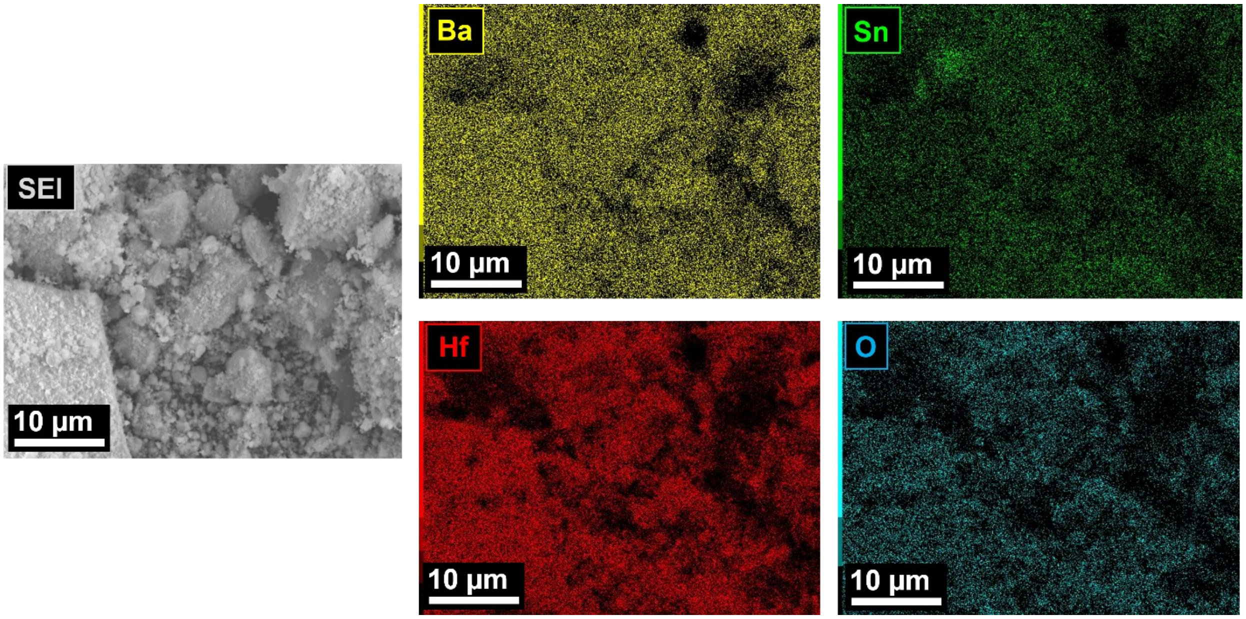


**Figure S6.** Bulk SEM and EDS elemental mapping for ~1 µm $BaHfO_3$ after Sn(II)-exchange $K_2SnCl_5$, with the high-resolution image (left), and elements (right) shown as Ba in yellow, Sn in green, Hf in red, and O in blue.

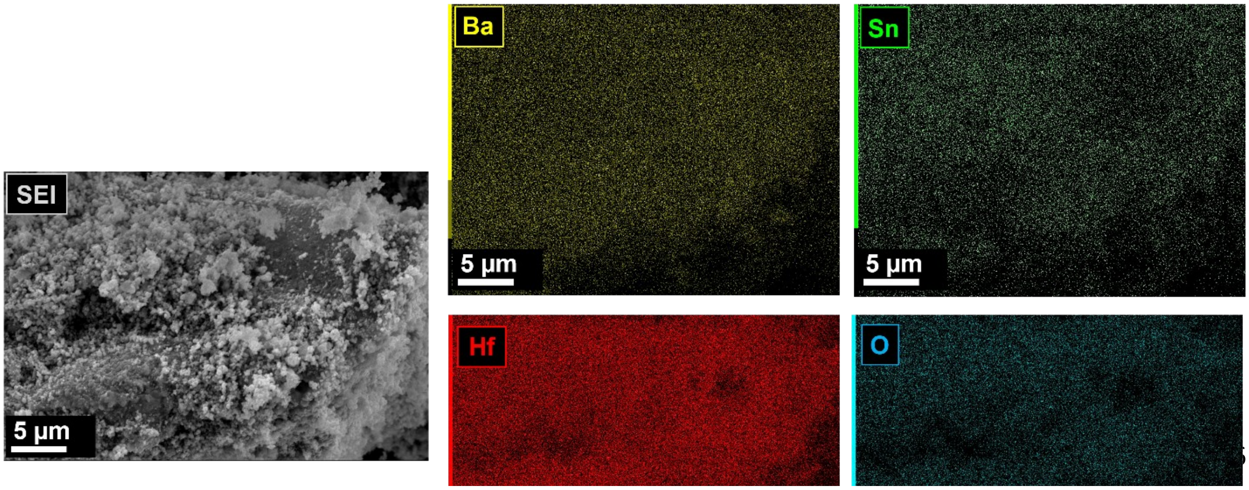


**Figure S7.** Bulk EDS elemental mapping for ~350 nm $BaHfO_3$ after Sn(II)-exchange $K_2SnCl_5$, with the high-resolution image (left), and elements (right) shown as Ba in yellow, Sn in green, Hf in red, and O in blue.

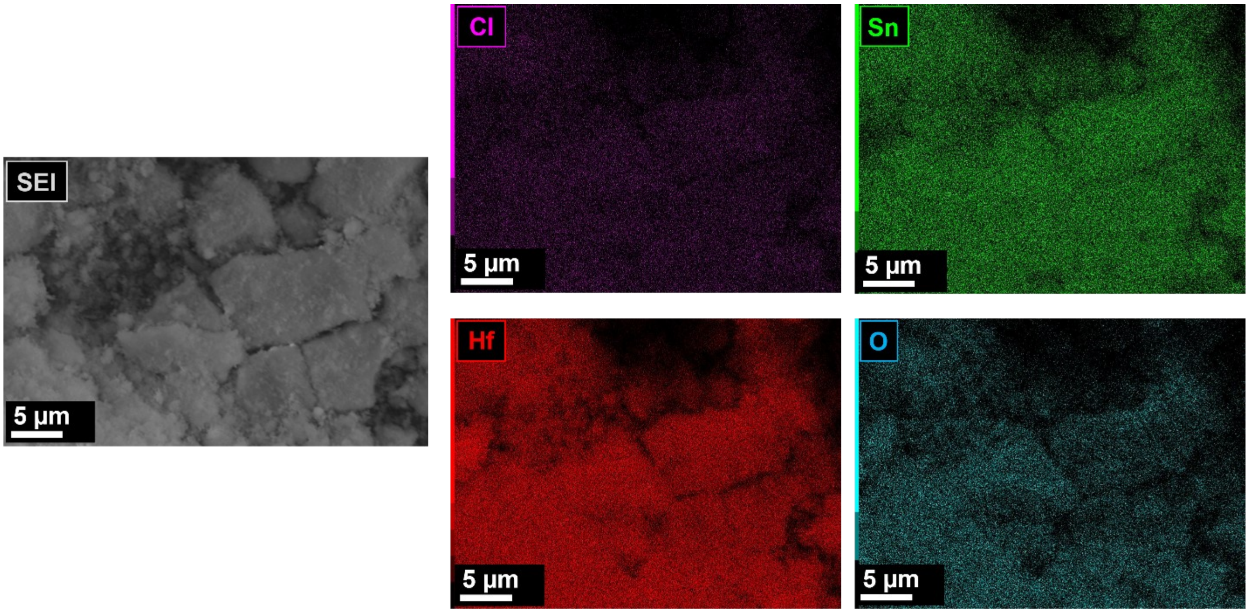


**Figure S8.** Bulk EDS elemental mapping for ~100 nm $BaHfO_3$ after Sn(II)-exchange $K_2SnCl_5$, with the high-resolution image (left), and elements (right) shown as Cl in purple, Sn in green, Hf in red, and O in blue. Ba was not detected (see Figure S8C).

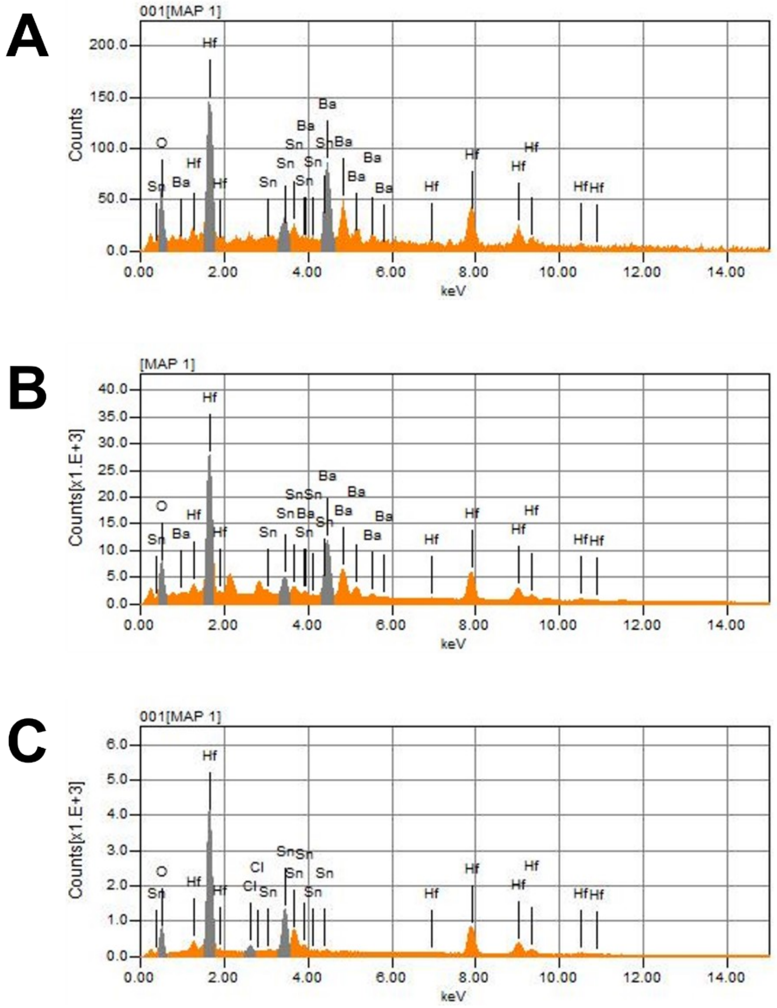


**Figure S9.** Bulk EDS spectra for $BaHfO_3$ with particle sizes of (A) ~1 μm, (B) ~350 nm, and (C) ~100 nm after Sn(II)-exchange with $K_2SnCl_5$. The first peak is the carbon support tape.
